# Supplementary material for: Cylindrical compression of thin wires by irradiation with a Joule-class short-pulse laser
Source: Nat Commun. 2024 Sep 12;15:7896. doi: 10.1038/s41467-024-52232-6 (PMC11392940; doi:10.1038/s41467-024-52232-6)
Supplement: Supplementary file 1 — Supplementary Information [file 41467_2024_52232_MOESM1_ESM.pdf]

Supplementary Information: Cylindrical compression of thin wires by irradiation with a Joule-class short pulse laser

Alejandro Laso Garcia[1†], Long Yang[1†], Victorien Bouffetier[2], Karen Appel[2], Carsten Baehtz[1], Johannes Hagemann[3], Hauke Höppner[1], Oliver Humphries[2], Thomas Kluge[1], Mikhail Mishchenko[2], Motoaki Nakatsutsumi[2], Alexander Pelka[1], Thomas R. Preston[2], Lisa Randolph[2], Ulf Zastrau[2], Thomas E. Cowan[1,4], Lingen Huang[1*], Toma Toncian[1*]

[1]Helmholtz-Zentrum Dresden - Rossendorf, Bautzner Landstraße 400, Dresden, 01328, Germany.
[2]European XFEL, Holzkoppel 4, Schenefeld, 22869, Germany.
[3]Deutsches Elektronen-Synchrotron DESY, Notkestraße 86, Hamburg, 22607, Germany.
[4]Technische Universität Dresden, Dresden, 01062,Germany.

*Corresponding author(s). E-mail(s): lingen.huang@hzdr.de; t.toncian@hzdr.de;
†These authors contributed equally to this work.

1 Supplementary Figures

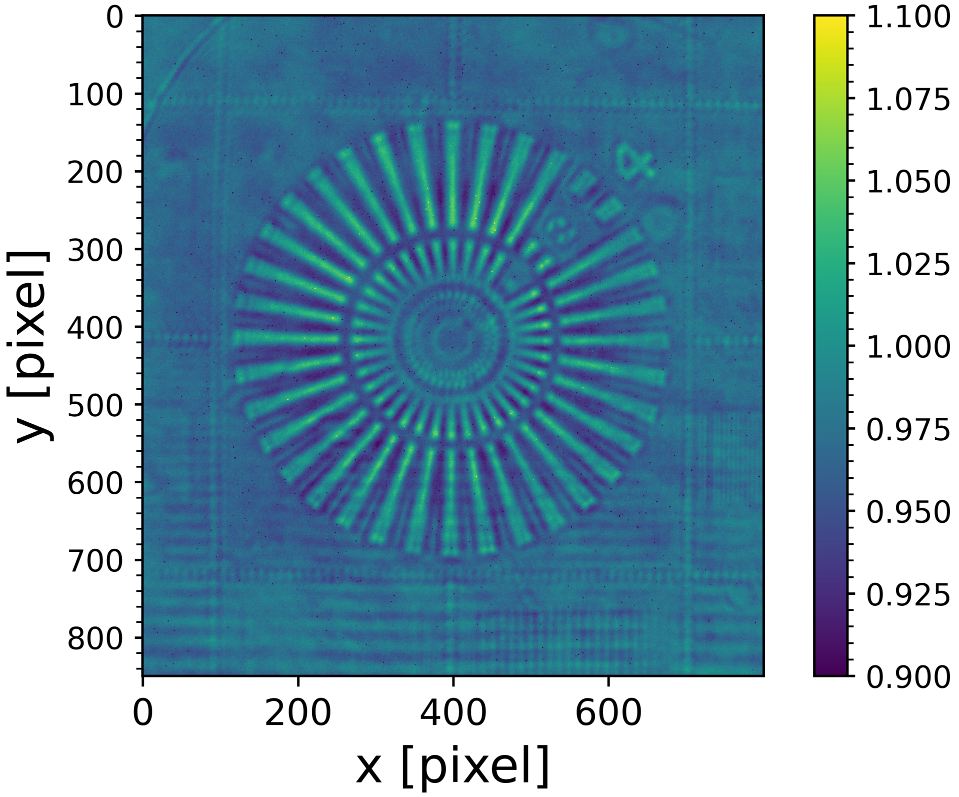

Fig. S1 X-ray imaging of a Siemens star calibration sample.

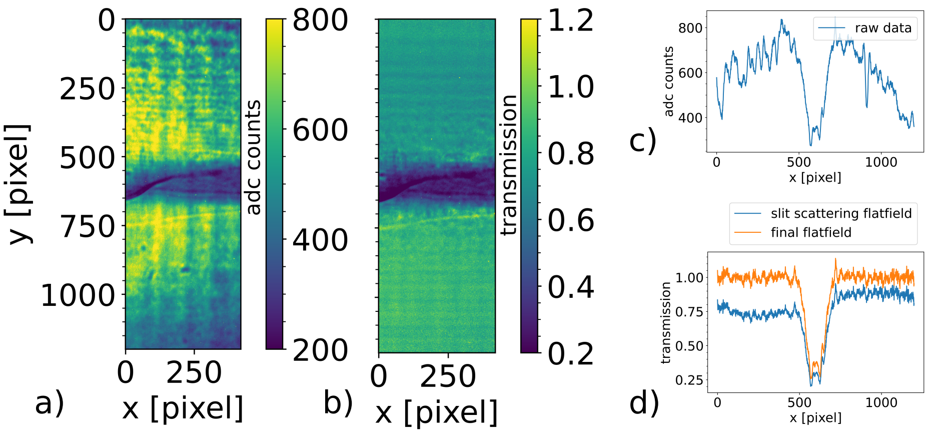

Fig. S2 Experimental data flatfielding steps: a) The raw data as measured by the detector. b) Flatfielded data reducing the slit scattering. c) Lineout of the raw data at a distance 42 µm from the laser interaction point. d) Lineout of the flatfield by the slit scattering and the final flatfielding by fitting the intensity outside the wire shadow.
